# Validation of a Smartwatch-Based Workout Analysis Application in Exercise Recognition, Repetition Count and Prediction of 1RM in the Strength Training-Specific Setting

**DOI:** 10.3390/sports9090118

**Published:** 2021-08-27

**Authors:** Katja Oberhofer, Raphael Erni, Mark Sayers, Dominik Huber, Fabian Lüthy, Silvio Lorenzetti

**Affiliations:** 1 Swiss Federal Institute of Sport Magglingen, Elite Sports, 2532 Magglingen, Switzerland; katja.oberhofer@baspo.admin.ch (K.O.); raphael.erni@unik-sports.com (R.E.); fabian.luethy@baspo.admin.ch (F.L.); 2 Department of Movement and Sport Sciences, University of Fribourg, 1700 Fribourg, Switzerland; 3 School of Health and Behavioural Sciences, University of the Sunshine Coast, Sunshine Coast, QLD 4556, Australia; MSayers@usc.edu.au; 4 Institute for Biomechanics, DHEST, ETH Zurich, 8093 Zurich, Switzerland; huber.dominik.sz@gmail.com

**Keywords:** resistance training, muscle strength, physical conditioning, smartphone, wearable electronic devices, biomechanics

## Abstract

The goal of this study was to assess the validity, reliability and accuracy of a smartwatch-based workout analysis application in exercise recognition, repetition count and One Repetition Maximum (1RM) prediction in the strength training-specific setting. Thirty recreationally trained athletes performed four consecutive sets of barbell deadlift, barbell bench press and barbell back squat exercises with increasing loads from 60% to 80% of their estimated 1RM with maximum lift velocity. Data was measured using an Apple Watch Sport and instantaneously analyzed using an iOS workout analysis application called StrengthControl. The accuracies in exercise recognition and repetition count, as well as the reliability in predicting 1RM, were statistically analyzed and compared. The correct strength exercise was recognised in 88.4% of all the performed sets (N = 363) with accurate repetition count for the barbell back squat (*p* = 0.68) and the barbell deadlift (*p* = 0.09); however, repetition count for the barbell bench press was poor (*p* = 0.01). Only 8.9% of attempts to predict 1RM using the StrengthControl app were successful, with failed attempts being due to technical difficulties and time lag in data transfer. Using data from a linear position transducer instead, significantly different 1RM estimates were obtained when analysing repetition to failure versus load-velocity relationships. The present results provide new perspectives on the applicability of smartwatch-based strength training monitoring to improve athlete performance.

## 1. Introduction

The research field of human activity recognition by means of commercially available, wearable technologies has gained an increasing focus in sports and health science for proactively monitoring and assisting users in their activities [1]. Wireless technologies, including Inertial Measurement Units (IMUs) and Global Positioning System (GPS) trackers, have become readily accessible in ubiquitous devices such as smartphones and smartwatches to monitor physical activity and performance in sports [2,3,4]. Thereby, the computational power of smartphones and smartwatches is ever increasing, with enhanced user interfaces that enable analysis of the wireless data in real time [5].

The application of wearable technologies to repetitive aerobic activities is well researched and successfully introduced to the market [1]; yet, their application to resistance training still remains limited [2]. In comparison to aerobic activities, such as outdoor running or cycling, performance monitoring of stationary strength training workouts requires careful consideration of sensor positioning and more advanced numerical analysis of the available data [3]. In addition, the execution diversity between exercises and individual athletes further complicates the analysis [4].

In an early effort to use smartphones for strength training monitoring, a dynamic time warping-based algorithm was introduced to identify exercise and count repetitions based on the available acceleration data [1,3]. The proposed numerical algorithm was tested both indoors with weight machines and for outdoor scenarios using free weights and resistance band exercises with promising results, i.e., below 1% classification error rate while remaining computationally inexpensive. In similar research, a prototypical machine learning algorithm was introduced for exercise recognition of three different strength exercises with dumbbells using a wrist-worn smartwatch with a demonstrated mean recognition rate of 97.7% in 20 adults [1,3]. More recent efforts led to the FitCoach, a virtual fitness coach to assess dynamic postures during workouts using data from wearables and smartphones, which was tested in 12 participants and 9 different strength exercises with an average exercise detection rate of 95% [5,6]. FitCoach was developed to combine exercise recognition and interpretation of wireless data into an easy-to-understand exercise review score for performance evaluation and recommendation to avoid injury [6]; however, no reference was made with regards to the One Repetition Maximum (1RM) as the key indicator of strength training performance.

The 1RM as ‘the maximum load that can be lifted through a full range of motion’ is known as the most valid indicator of an individual’s dynamic strength [7], and thus, the quantification of an individual’s 1RM is fundamental in the design of safe and effective resistance training programs [8]. The direct assessment of 1RM is time-consuming and depends on the athlete’s experience, motivation and fatigue, with risk of musculoskeletal injury due to maximum loading [9]. In contrast, indirect methods have been introduced to predict the 1RM based on well-established linear regression techniques, including the repetition to failure method [10,11] as well as the relationship between load and lifting velocity (L-V relationship) [7,12,13,14,15]. In order to derive the L-V relationship for individual athletes and exercises, commercially available Linear Position Transducers (LPT) are generally used [16,17]. Yet, the application of LPT devices to free weight and sport-specific strength exercises is compromised. In particular, LPT devices are limited in picking up fluctuations in lifting velocities due to horizontal or asymmetrical displacements depending on the positioning and manufacturer of the device [7].

Recent advances in smartwatch-based technologies hold great potential to help improve 1RM predictions for strength exercises without Smith machines in the strength training-specific setting. Towards this goal, Lorenzetti and Huber [18,19] introduced an iOS workout analysis application for the Apple Watch called StrengthControl to determine exercise recognition and repetition count, and, piloting towards the prediction of 1RM, muscle loading and fatigue. The StrengthControl app was tested in one subject for five resistance training exercises (barbell biceps curl, barbell bench press, barbell back squat, dumbbell lateral raise, and dumbbell biceps curl with twist), with a reported mean error in exercise recognition of 3.5% and 0.92% in repetition counting, respectively [18]. However, no study has yet to report on the reliability and accuracy of smartwatch-based measurements in predicting 1RM outside of the research setting. The goal of this study was to assess the validity, reliability and accuracy of the iOS StrengthControl app in exercise recognition, repetition count, and 1RM prediction in recreational athletes in the strength training-specific environment.

The present results suggest that further investigations are needed to improve the accuracy of the velocity estimates from smartwatch-based readings to predict 1RM. A reduction in technical errors and time lag in data transfer may be achieved by accounting for subject-specific body height and range of motion, as well as giving clear instructions on pauses between concentric and eccentric movement phases. Future research should also consider alternative motion sensors or vision-based methods for human activity recognition to assess an individual’s 1RM in the strength training-specific setting.

## 2. Materials and Methods

### 2.1. Study Design

**Figure 1:**
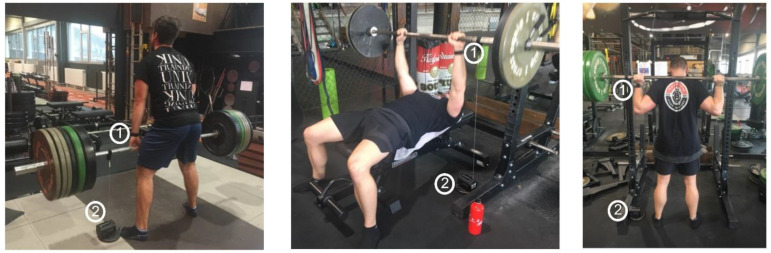
Measurement devices used during testing: (1) Apple Watch Sport, (2) GymAware PowerTool.

Thirty physically healthy, recreationally trained athletes performed four consecutive sets of barbell deadlift, barbell bench press, and barbell back squat exercises with increasing loads from 60% to 80% of their estimated 1RM. The focus on each lift was to maximize lift velocity. The loading regime was chosen to enable the indirect prediction of 1RM via the L-V relationship. The acceleration of the left wrist and the velocity of the barbell were simultaneously measured during all repetitions for all exercises using the Apple Watch Sport on the participant’s left wrist and an LPT (GymAware PowerTool) strapped around the bar, respectively (Figure 1). The GymAware PowerTool is an optical encoder LPT device that uses an optical encoder with infrared light for distance-based sampling. Exercise recognition and repetition count were derived using the iOS StrengthControl app [18]. 1RMs were predicted based on the repetition to failure method [10], as well as using two reported regression equations for the L-V relationship [13,14]. The accuracies in exercise recognition and repetition count were statistically analysed, and 1RM estimates were compared by means of correlation analysis.

### 2.2. Participants

All participants were physically healthy (mean age 28.4 ± 6.0 years) with a heterogeneous history of strength training (mean 4.8 ± 3.9 years), participating in their own strength training programs one to four days a week. The physical characteristics of the participants are given in Table 1. The participants were recruited via both online advertisement and email. Potential participants were excluded if they were experiencing acute or chronic musculoskeletal pain, had a musculoskeletal surgery within the last 12 months, or ongoing rehabilitation or treatment of musculoskeletal complaints, injury or disease. The study protocol adhered to the Declaration of Helsinki and was approved by the local ethics committee. At the outset of the study, participants were informed of the study protocol, the schedule, the nature of the exercises and measurements to be taken before signing an informed consent form.

### 2.3. Instruments and Exercise Equipment

The StrengthControl app was previously introduced and tested in one subject [18]. The iOS and watchOS application, Xcode (Apple Inc., Cupertino CA 95014, USA), incorporates a human activity recognition algorithm by FocusMotion to analyse the accelerometer data of the Apple Watch with demonstrated high functionality. An integrated user interface for the iPhone enables the real-time classification and presentation of the measured data for different strength exercises. Importantly, the StrengthControl app offers a user-defined weight insert option to enable the estimation of 1RM in addition to exercise recognition and repetition count.

For the measurements, an Apple Watch Sport (1st generation) was strapped around the participant’s left wrist and connected to an iPhone 6s with iOS 11.4.1 installed. The StrengthControl app was developed for the 1st generation Apple Watch Sport, which was the reason for choosing this device. The GymAware PowerTool was attached with a Velcro strap around the bar according to the instructions of the manufacturer to ensure that a perpendicular angle was achieved during all lifts and was then paired through Bluetooth to an iPad Air. Both the iPhone and the iPad had the required software installed for data acquisition (GymAware, v2.5.1, c2014-18, Kinetic Performance Technology Pty Ltd., Mitchell, ACT 2911, Australia, and StrengthControl, v1.8, Betatester). For the resistance training, a standard Olympic barbell (20 kg = 44 lbs) and weight plates (5–20 kg = 11–44 lbs) were used.

### 2.4. Procedure

The data was collected in the training-specific setting of the participants at three different gym facilities. Participants were asked to refrain from strength training at least 48 h before the testing. Each participant performed an individual 10-min warm up session of aerobic exercises on the rower or bike, followed by a warm up set of the strength exercises with minimal weights. For data acquisition, all subjects performed four consecutive sets of barbell deadlift, barbell bench press and barbell back squat with increasing loads from 60 to 80% of their estimated 1RM based on training experience. The aim of loading was to enable no more than 10 repetitions until failure [20]. If a participant estimated his/her 1RM too low, an additional fifth set was performed to ensure fatigue within 10 repetitions. Subjects were allowed an adequate rest of 3–5 min between each set [21]. Instructions on exercise execution were given prior to testing according to established guidelines [22]. In particular, participants were instructed to execute each exercise as fast as possible to enable the prediction of 1RM based on the L-V relationship.

### 2.5. 1RM Prediction

Three different equations to estimate the 1RMs of each subject for the barbell bench press, the barbell back squat, and the barbell deadlift were adopted [10,14,15] (Table 2). One equation was based on the repetition to failure method [10], and two of the equations were based on well-established linear regression techniques to derive the 1RM based on the L-V relationship [13,14]. Thereby, the L-V equation proposed in Sayers, Schlaeppi, Hitz and Lorenzetti [15] is based on findings that the peak vertical bar velocity yields more accurate predictions of Smith Machine bench press 1RM than mean bar velocity.

The calculation of 1RM based on the L-V relationships required the definition of the minimum velocity threshold (MVT) as “the mean concentric velocity produced on the last successful repetition of a set to failure performed with maximal lifting effort” [14]. The MVT was set at 0.15 for the barbell bench press, 0.25 for the barbell back squat and 0.3 for barbell deadlift, respectively. MVT values were set according to reported values in the literature for recreationally trained athletes but not specifically powerlifters, who tend to show lower MVT values [7,13,22,23,24,25].

### 2.6. Data Analysis

Data was sampled in real-time through the iPad Air from the LPT GymAware PowerTool, as well as through the iPhone 6s from the Apple Watch Sport, and sent to a MacBook Pro via Bluetooth for storage, analysis and presentation of results.

Two-sample paired t-tests were used to analyse the significance of the differences between the predicted values from the StrengthControl app and the actual values of repetition count, as well as paired 1RM estimates from the three different prediction algorithms (Table 2). Thereby, the Root Mean Square Error (RMSE) was calculated for each exercise and each set as follows:

$$\mathrm{RMSE}=\sqrt{\bar{{\left(p_{1}-p_{2}\right)}^{2}}}$$

where *p*_1_ is the actual value and *p*_2_ the predicted value, or two predicted values from two different 1RM prediction equations, respectively.

For each strength exercise, linear regression analysis was done between paired predicted 1RMs from the three different equations (Table 2), and the coefficients of determination *R^2^* of the linear regression lines were derived. The reliabilities of the estimates from linear regression analysis were further interpreted using Pearson correlation coefficients, and described as trivial (0.0–0.1), low (0.1–0.3), moderate (0.3–0.5), high (0.5–0.7), very high (0.7–0.9), or practically perfect (0.9–1.0) [26]. The level of significance was set at *p* = 0.05 for all statistical tests.

## 3. Results

### 3.1. Exercise Recognition and Repetition Count

The accuracies of the StrengthControl app in exercise recognition and repetition count are shown in Table 3 and Table 4. Overall, the correct strength exercise was recognised in 88.4% of all the performed sets (N = 363). The barbell bench press was recognised with the highest accuracy of 96.5%, followed by the barbell deadlift with 92.2% and the barbell back squat with 76.5%, respectively. The results for the barbell bench press and barbell deadlift are in line with previously reported accuracies of wearable technologies in exercise recognition (i.e., 97.7% [1], 95% [6], 96.5% [18]). The inaccuracies in the recognition of the barbell back squat may be explained by the inhomogeneous group of participants presenting with large differences in body height and range of motion (Table 1) as well as technical difficulties in data transfer, with 13 of 121 sets of the barbell back squat being Nill (i.e., non-detectable) and 10 sets being falsely detected. The difference between the predicted repetition count and the actual repetition count for the correctly recognised sets was insignificantly small for the barbell back squat (*p* = 0.68), and acceptable for the barbell deadlift (*p* = 0.09); however, repetition count for the barbell bench press was poor (*p* = 0.01), Table 4.

### 3.2. 1RM Predictions

**Figure 2:**
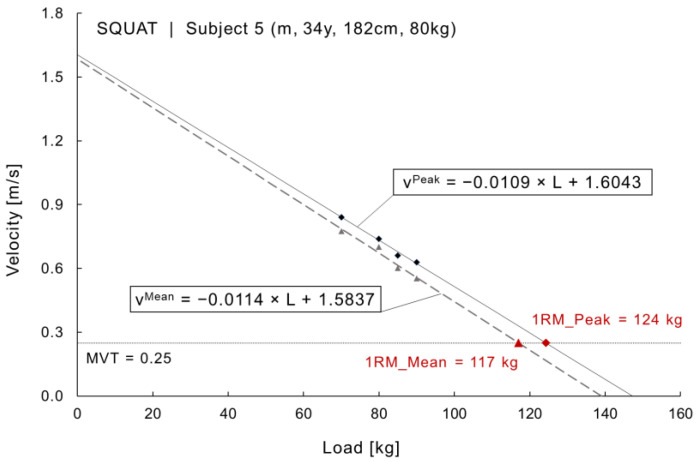
Calculation of 1RM_Mean and 1RM_Peak (Table 2) based on the measured lifting velocity. Graph includes the linear regression lines for the measured mean and peak velocities from the LPT (v^Mean^, v^Peak^), with an assumed minimum velocity threshold of MVT = 0.25 for the barbell back squat.

Only 8.9% of attempts to predict 1RM using the StrengthControl app were successful (Table 5). Instead, the LPT data from the GymAware PowerTool was used for the calculation and comparison of the 1RM prediction equations (Table 2). The results from the correlation analysis between 1RM predictions using the LPT data for the three strength exercises are listed in Table 6. The L-V relationship of one subject is shown in Figure 2 to exemplify the prediction of 1RM_Mean and 1RM_Peak based on the empirical relationship between load and measured lifting velocity.

The resulting 1RM predictions from the three different algorithms were significantly different in all paired comparisons except for the comparison between 1RM_Peak and 1RM_Mean for the barbell deadlift (*p* = 0.68, Table 6). The reliability of the estimates from linear regression analysis was nearly perfect for the bench press exercise (Pearson’s r = 0.99) and very high for the barbell back squat (r = 0.89–0.96) and the barbell deadlift (r = 0.84–0.90).

Initially, the MVT values for the calculation of 1RM_Mean and 1RM_Peak were set at 0.15 for the barbell bench press, 0.25 for the barbell back squat, and 0.3 for the barbell deadlift, respectively. Following data acquisition, MVT values for the present study group were retrospectively calculated. The study-specific MVT values were 0.16 ± 0.05 for the barbell bench press, 0.35 ± 0.04 for the barbell back squat, and 0.45 ± 0.13 for the barbell deadlift, respectively.

The resulting MVT value for the barbell bench press is comparable to previously reported MVT values in similar subject groups (i.e., recreationally trained athletes), such as 0.15 ± 0.03 [22], 0.16 ± 0.04 [12,27], and 0.17 [28]. Lower MTV values for the same exercise are reported in the literature for athletes with increased level of strength training experience, such as powerlifters with a reported MVT of 0.10 ± 0.04 [24] and college-age experienced benchers with an MVT of 0.14 ± 0.04 [29]. The resulting MVT for the barbell back squat can only be compared with the results in [25] that reported an MVT of 0.37 for paused squats, and 0.39 for regular squats using a Smith machine; while MVT values for the barbell deadlift were previously reported to be 0.14 ± 0.05 in experienced powerlifters, which is significantly smaller compared to the present results. The significant difference may again be attributed to the contrasting level of sports performances and experience in lifting. Indeed, it becomes apparent from the L-V relationship as shown in Figure 2 that a decrease in MVT would result in an increase in the calculated load at 1RM and vice versa.

## 4. Discussion

Inaccuracies in exercise recognition and repetition count, as well as failed attempts to predict 1RM using the StrengthControl app, can largely be explained by inter- and intra-subject differences in exercise execution within and between sets, as well as technical difficulties with the smartwatch not being able to capture and process the data correctly. In order to execute the strength exercises with maximal concentric velocity, the participants performed rapid movements without any instructions regarding the pauses between the concentric and eccentric phase of each repetition. It was previously suggested that imposing a pause between eccentric and concentric movements would increase the reliability of acceleration measurements using a smartwatch [30]. Thus, it is possible that clear instructions to the pauses may have helped to lower the coefficient of variation in the smartwatch data readings, thereby increasing the accuracy in exercise recognition, repetition counting and successful attempts to predict 1RM.

Technical difficulties and disturbances arose in the wireless transfer of data from the smartwatch to the smartphone. Unfortunately, the smartwatch ended up either stuck in a loop, or not all data was transmitted due to a lag in transmission. The lag was likely caused by the slow processer that is embedded in the first generation of the Apple Watch Sport. Here, a newer model of the Apple Watch may have helped to eliminate problems with wireless data transfer. However, similar research also reported that smartphone-based accelerometers presented with a considerable loss of data that was not correctly detected by the sensor during bench press exercises with the Smith machine [17]. In contrast to accelerometers that are specifically built for high-velocity measurements with sampling frequencies of 200 to 500 Hz, accelerometers embedded in the smartwatch or smartphone remain low-cost and based on low frequency sampling that is not precise enough to analyse explosive movement and repetitive movements at higher velocities.

In comparison to direct 1RM assessment, the prediction of 1RM based on the L-V relationship can be done on a regular basis without the high risk of injury associated with maximal loading. Indeed, previous findings suggest that there is no need to test overly heavy loads, as the prediction of 1RM from the L-V relationship derived at sub-maximal loads with exercise execution at maximal velocity is just as accurate [13,14,15]. Yet, the key challenge in the prediction of 1RM based on the L-V relationship is the requirement for accurate velocity measures during exercise performance, which were not shown to be reliable enough using the proposed methodology. Here, Peláez Barrajón and San Juan [17] also concluded that smartphone-based accelerometers are less reliable for the measurement of concentric mean velocity during bench press exercises compared to LPT devices. It was suggested that accurate measures of range of motion and body height are required to improve the accuracy in the calculation of velocity parameters using the smartwatch [17]. Unfortunately, subject-specific differences in body height and range of motion could not be accounted for in the StrengthControl app, likely contributing to some of the inaccuracies in the present results. Furthermore, participants may have performed strength exercises with submaximal velocity even though maximal velocity is required to adequately calculate 1RM using the L-V relationship. Thus, the option to calculate 1RM using the repetition to failure method [10,11] should also be considered for implementation into any workout analysis application using wearable technologies.

As an alternative to IMUs and GPS trackers for performance tracking, research in human activity recognition has been directed towards 3D pose estimation using data from the high-speed camera in smartphones in combination with advanced image analysis and deep learning techniques in computer vision [31,32,33]. These advances in computer vision provide alternative, and possibly complementary, means to assess lifting velocity during strength exercises for predicting 1RM. Here, the so-called *PowerLift* application for the iOS was recently introduced to measure barbell velocity by video-recording the lift using an iPhone [32,33]. It was demonstrated that the *PowerLift* application achieved accurate and reliable mean barbell velocity measures during the full squat, bench press and hip thrust exercises when compared with the results from an LPT device [32]. Furthermore, a method was introduced that combined a single hand-held camera and a set of 13 IMUs attached to the body limbs to estimate 3D pose in the wild [34]. While the use of 13 smartwatch-based IMUs is not feasible for widespread application, combining smartwatch-based and iPhone-based readings with advanced deep learning techniques seems promising to open new perspectives for the advancement of strength training monitoring.

Two limitations of the present study that haven’t been discussed are the heterogeneity of participants, as well as the lack of directly assessing each participant’s 1RM for comparison with the adopted 1RM equations as the gold standard. The study group was chosen to represent potential end-users of the StrengthControl app who are common in the recreational strength training-specific setting. However, a more confined study group, for example focusing on experienced power-oriented athletes of similar age and gender, would have likely led to reduced inaccuracies in results due to smaller inter- and intra-subject differences in exercise execution. Unfortunately, directly assessing 1RM in the present study group was not feasible due to the study design, time constraints and experience of the participants. Here, power athletes may be more willing and experienced with direct 1RM testing, and should be considered for further validation of 1RM predictions from wearable and smartphone-based technology in future work.

## 5. Conclusions

Further investigations are needed to improve the accuracy of the velocity estimates from smartwatch-based readings for predicting 1RM. Future research may account for subject-specific body height and range of motion, and possibly use different accelerometers and operating systems with clear instructions on pauses between concentric and eccentric movement phases in order to reduce technical errors in data transfer and time lag. Alternatively, advanced methods in computer vision for video-based analysis using smartphones may provide new perspectives to assist with the accurate assessment of 1RM for improved strengths training monitoring.

## Figures and Tables

**Table 1:** Physical characteristics (Mean ± Standard Deviation (SD)) of the total subject group, as well as for men and women separately, including the Body Mass Index (BMI) and their estimated 1RM for the barbell bench press (BBP), the barbell back squat (BBS), and the barbell deadlift (BDL).

Variable	Total (*n* = 30)	Men (*n* = 14)	Women (*n* = 16)
Age [years]	28.4 ± 6.0	28.1 ± 6.5	28.6 ± 5.8
Height [cm]	1.72 ± 0.09	1.79 ± 0.06	1.67 ± 0.07
Weight [kg]	72.3 ± 15.7	84.6 ± 13.2	61.9 ± 8.1
BMI	24.1 ± 3.4	26.5 ± 3.3	22.0 ± 1.8
Years of training experience	4.8 ± 3.9	6.8 ± 4.8	3.1 ± 1.7
1RM^BBP^ [kg]	70.6 ± 34.5	96.5 ± 36.9	43.9 ± 7.6
1RM^BBS^ [kg]	116.0 ± 42.2	143.2 ± 50.6	84.8 ± 19.9
1RM^BDL^ [kg]	126.5 ± 47.7	154.3 ± 61.1	94.3 ± 20.4

**Table 2:** Overview of the three 1RM prediction equations and abbreviations used.

Abbreviation	Ref	Formula
1RM_Rep	Brzycki [10]	L^1RM_REP^ = L× (36/(37 − rep))
1RM_Mean	Jovanović and Flanagan [14]	L^1RM_Mean^ = (MVT − intercept^VMean^)/slope^VMean^
1RM_Peak	Sayers, Schlaeppi, Hitz and Lorenzetti [15]	L^1RM_Peak^ = (MVT − intercept^VPeak^)/slope^VPeak^

Notes. L = load [kg]; rep = number of repetitions; VMean = average bar velocity; VPeak = peak bar velocity; slope = linear L-V regression line; intercept = linear L-V regression line; MVT = minimum velocity threshold.

**Table 3:** Exercise recognition accuracy for each set (N) of the three strength exercises, with TRUE being the correctly recognized exercise, FALSE the wrongly recognized exercise and NILL meaning no exercise was detected.

Exercise	N	TRUE	FALSE	NILL	%TRUE
Barbell bench press	119	115	3	1	96.5%
Barbell back squat	121	98	10	13	76.5%
Barbell deadlift	124	115	4	5	92.2%
Total	363	327	17	19	88.4%

**Table 4:** Accuracy in repetition count for each set (N) of the correctly recognized strength exercises, with the root mean square error (RMSE), p-value and Pearson’s correlation coefficient between the true repetition count (TR) and the recognized repetition count (RR).

Exercise	N	TR_Mean_	RR_Mean_	RMSE	*p*-Value	Pearson
Barbell bench press	115	8.84 ± 2.01	9.44 ± 3.15	1.36 ± 2.16	0.01	0.61
Barbell back squat	98	9.47 ± 1.20	9.31 ± 4.09	2.51 ± 3.08	0.68	0.24
Barbell deadlift	115	9.41 ± 1.54	9.97 ± 3.80	2.57 ± 2.47	0.09	0.37
Total	327	9.23 ± 1.59	9.58 ± 3.67	2.14 ± 2.56	0.06	0.40

**Table 5:** 1RM predictions using the StrengthControl app for the three strength exercises based on the measured data of all subjects (N = 30).

Exercise	N	Attempts	Predicted	% Success
Barbell bench press	30	30	2	6.7%
Barbell back squat	30	30	1	3.3%
Barbell deadlift	30	30	3	10%
Total	90	90	6	8.9%

**Table 6:** Results from statistical analysis of 1RM predictions between the three different algorithms (Table 1) for the barbell bench press, the barbell back squat and the barbell deadlift, including coefficient of determination (R^2^) from linear regression analysis, root mean square error (RMSE), *p*-value and Pearson’s correlation coefficient.

Exercise	Paired Algorithms	R^2^	RMSE	*p*-Value	Pearson
Barbell bench press	1RM_Mean, 1RM_Rep	0.9799	4.63	<0.01	0.99
	1RM_Peak, 1RM_Rep	0.9791	4.00	0.04	0.99
	1RM_Peak, 1RM_Mean	0.9846	5.10	<0.01	0.99
Barbell back squat	1RM_Mean, 1RM_Rep	0.8129	13.86	<0.01	0.90
	1RM_Peak, 1RM_Rep	0.7919	19.76	<0.01	0.89
	1RM_Peak, 1RM_Mean	0.9163	11.41	<0.01	0.96
Barbell deadlift	1RM_Mean, 1RM_Rep	0.6540	22.71	<0.01	0.89
	1RM_Peak, 1RM_Rep	0.7078	21.82	0.01	0.84
	1RM_Peak, 1RM_Mean *	0.8094	16.90	0.68	0.90

* No significant difference between methods detected (i.e., *p* > 0.05).
